# The Reality of Patient-Reported Outcomes of Health-Related Quality of Life in an Italian Cohort of Patients with Inflammatory Bowel Disease: Results from a Cross-Sectional Study

**DOI:** 10.3390/jcm9082416

**Published:** 2020-07-28

**Authors:** Tiziana Larussa, Danilo Flauti, Ludovico Abenavoli, Luigi Boccuto, Evelina Suraci, Raffaella Marasco, Maria Imeneo, Francesco Luzza

**Affiliations:** 1 Department of Health Sciences, University of Catanzaro “Magna Graecia”, 88100 Catanzaro, Italy; larussa@unicz.it (T.L.); daniloflauti@hotmail.it (D.F.); l.abenavoli@unicz.it (L.A.); e.suraci@libero.it (E.S.); raffaellamarasco1@gmail.com (R.M.); graziaimeneo@hotmail.it (M.I.); 2 Greenwood Genetic Center, Greenwood, Clemson University, Clemson, SC 29631, USA; lboccuto@ggc.org

**Keywords:** inflammatory bowel diseases, quality of life, Crohn’s disease, ulcerative colitis, quality of care

## Abstract

Inflammatory bowel disease (IBD) has a negative impact on patients’ physical and psychological well-being, social performance, and working capacity, thereby worsening their health-related quality of life (HRQoL). Clinicians should take care of the patients’ global health, including the psychological, social, and emotional spheres. We aimed to investigate the reality of patient-reported outcomes of HRQoL in a series of IBD patients. Consecutive Crohn´s disease (CD) and ulcerative colitis (UC) patients in clinical remission were recruited. The survey consisted of the Short Inflammatory Bowel Disease Questionnaire (S-IBDQ), the Hospital Anxiety and Depression Scale (HADS), the Brief Illness Perception Questionnaire (B-IPQ), and a questionnaire dealing with impact of IBD on patients’ lives. Demographic and clinical characteristics were recorded. Of 202 participants (29% CD and 71% UC; 54% male; median age 48 years; mean disease duration 14 ± 11 years), 52% had poor HRQoL, 45% anxiety/depression, and 35% sleep disturbance and a high perception of disease (mean score 42.8 ± 14.3). In the multivariate analysis, a low HRQoL was rather associated with UC than CD (*p* = 0.037), IBD surgery (*p* = 0.010), disease duration (*p* = 0.01), sleep disturbance (*p* = 0.014), anxiety/depression (*p* = 0.042), and high illness perception (*p* = 0.006). IBD affected working performance and social activities in 62% and 74% of patients, respectively. Satisfaction regarding quality of care, biologics, and surgery approach were claimed in 73%, 69%, and 76% of patients, respectively. Although 84% of patients trusted their gastroenterologist, only 66% of them discussed IBD impact on HRQoL during visit. In a series of IBD patients in remission, the low HRQoL was significantly associated with surgery, disease duration, sleep disturbance, anxiety/depression, and high illness perception. Even though patients were satisfied with the quality of their care, it appears that clinicians should pay more attention to patients’ emotional status.

## 1. Introduction

Crohn’s disease (CD) and ulcerative colitis (UC) are the main types of inflammatory bowel disease (IBD), a collective term indicating a group of chronic intestinal disorders characterized by alternating periods of remission and relapse. Common symptoms are abdominal pain, diarrhea, fever and rectal bleeding, and frequent complications including abscesses, fistulas, and stenosis [1]. IBD represents a life-long disease which might require daily medication, frequent doctor’s appointments, and possible hospitalizations or surgery in a considerable number of patients [2]. Furthermore, in light of increasing treatment options, the safety issue of short- and long-term biological treatment should not be underestimated [3]. Consequently, IBD has a negative impact on patients’ physical and psychological well-being, social performance, and working capacity [4]. Today, quality of life represents an important aim of medical treatment and more generally of healthcare management [5]. In the case of chronic diseases like IBD, it is preferable to talk about health-related quality of life (HRQoL), which can be defined as the value assigned to the lifespan, taking into account the influence of limitations, changes in functional capacities, alterations of subjective perceptions, and impediments to social opportunities that an illness can determine [6]. Particularly during the phases of active disease, IBD patients report a strong impairment of HRQoL, dealing with anxiety, depression, sleep disturbance, and fatigue [7]. Although there was a strong confirmation that HRQoL is poorer during active disease, this condition frequently persists during the quiescent phases of the disease [8]. This means that mental and social well-being should be achieved beyond the absence of disease or physical infirmity and clinicians should take care of the patients’ global health, including the assessment of the psychological, social, and emotional spheres during routine visits [9]. Several studies have shown that IBD patients are more likely to suffer from depression and anxiety [10]. Although the exact relationship remains unclear, bi-directional processes have been suggested, as the experience of the disease is stressful enough to trigger or intensify an underlying psychiatric condition; conversely, anxiety or depression may themselves worsen HRQoL [11]. Perceived stress can also have a direct impact on the neurological regulation of intestinal motility and pain perception and, of note, it has been shown to promote direct effects on immune function [12]. Particularly in younger patients, poor HRQoL is often associated with negative illness perception, which is the subjective meaning that patients give to their disease [13]. Another important aspect is the patients’ satisfaction with treatment and trust in their healthcare providers, both factors that could affect compliance with the therapy and follow-up visit program and represent a challenging issue for gastroenterologists [14].

The aim of this study was to focus on the reality of patient-reported outcomes of HRQoL in patients with quiescent IBD, in order to assess factors which may affect their HRQoL.

## 2. Materials and Methods

### 2.1. Patients

From March 2019 to March 2020, a cross-sectional study was conducted at the U.O. of Pathophysiology of the Digestive System of the University Magna Graecia of Catanzaro (Catanzaro, Italy), in which patients with IBD in clinical remission were enrolled in order to assess their perceptions and unmet needs related to their HRQoL. The inclusion criteria for the study were a previous diagnosis of CD or UC, based on objective examination, patient’s clinical history, hemato-clinical tests, endoscopy with biopsies, and histological assessment. Disease activity was measured by the Harvey Bradshaw index (HBI) [15] for CD or by the Mayo Score (MS) [16] for UC. Patients were considered in remission and therefore eligible for the study if they showed an HBI < 5 for CD or a partial MS ≤ 2, with no subscore >1, for UC. Patients with a diagnosis of indeterminate colitis, those who had undergone major surgery in the last three months, or those who had suffered from severely disabling concomitant diseases (i.e., severe cardiopulmonary, renal or hepatic disease, Alzheimer’s disease, post-stroke sequelae) were excluded. Each patient was interviewed by the trained clinical team, and information regarding demographic characteristics, IBD, personal and family history, lifestyle, type of employment, level of education, previous relevant medical history, current co-morbidity, and the type of medications used was collected. Self-reported height and weight were used for the calculation of body mass index (BMI).

### 2.2. Interventions

Based on the inclusion and exclusion criteria, IBD outpatients who came consecutively to the unit to be evaluated periodically by follow-up visits were invited to enter a waiting room, where they were told the purpose of the study and how to participate. Patients who agreed to take part in the study gave informed consent and received a questionnaire to be filled in on their own. A member of the medical staff remained at their disposal in case of doubts or uncertainties in the filling in or if the patient encountered difficulties in completing the task. The questionnaire was anonymous, but there was a code for further analysis of the data. Participants were assured that the data would remain confidential.

### 2.3. Questionnaire

The survey consisted of the Short Inflammatory Bowel Disease Questionnaire (S-IBDQ) [17], the Hospital Anxiety and Depression Scale (HADS) [18], the Brief Illness Perception Questionnaire (B-IPQ) [19], and a series of questions regarding the impact of the disease on the life of the patient (Supplementary Materials File S1) [20]. The survey was planned to be self-administered and took around 20 min to be completed. Considering that the questionnaires S-IBDQ, HADS, and B-IPQ were translated from English into Italian, the entire questionnaire was administered to 20 volunteers before the enrolment of the patients in order to evaluate its feasibility and clarity. Then, the validity of the content and its reproducibility were verified with the help of a concomitant interview with the volunteers by the medical staff and a comparison between the two methods (κ = 0.70).

S-IBDQ is a tool used to evaluate HRQoL in patients with IBD, measuring their physical, social, and emotional states. It consists of 10 questions; to each of them, the patient could give a score from 1 to 7, with a total score between 10 and 70. HRQoL is considered to be slightly, moderately, or severely impaired for scores of 60–70, 45–60, and 10–45 points, respectively. Therefore, in our cohort, we defined the cut-off for relevant impairment of HRQoL as < 50 points. The aim of the HADS questionnaire is to measure the symptoms of anxiety and depression. It consists of 14 questions, seven of which are used to assess anxiety and seven for depression. Each question presents four alternatives with a scale of values from 0 to 3, in order to have an optimal balance between sensitivity and specificity; a value of 7 has been chosen as cut-off, so that the test is positive for values higher than 7 for both the anxiety and depression sub-scales. The B-IPQ is a questionnaire consisting of 9 items. Five items assess the cognitive perception of the disease, two items assess the emotional perception, and one item assesses the understanding of the disease. The ninth item is a causal item, where patients are asked to write down three self-assumed causes of their disease, and it was not applied for the current study. All the items, except for the causal question, are rated using a 0-to-10 response scale. For items 1, 2, 5, 6, and 8, a 0 score indicates a good disease perception, and a score of 10 indicates a bad disease perception. For items 3, 4, and 7, a score of 0 indicates a bad disease perception, and 10 indicates a good disease perception. To compute the score, it is necessary to reverse the score of items 3, 4, and 7 and add these to items 1, 2, 5, 6, and 8. In our study, the result was expressed as an overall score which represents the degree to which the illness is perceived as threatening or benign. A higher score reflects a higher perception of the disease. The final part of the survey consisted of 9 questions with the objective of examining the impact that the disease has on the working activities and social life of the patients and their perceptions and unmet needs in relation to the quality of care. It also included a question dedicated to the quality of sleep. Except for the question regarding sleep quality, these final questions were to be answered considering the entire course of the disease and not only the remission period.

### 2.4. Ethical Considerations

The study was conducted in accordance with the Helsinki Declaration. The patients received oral and written information about the study. All participants were informed that participation was voluntary and that they could withdraw at any time without consequences. The study protocol was approved by the local Research Ethics Committee (n.86/2019), and written informed consent was obtained from all participants.

### 2.5. Statistical Analysis

The Kolmogorov–Smirnov test of normality was used to analyze the data distribution. Continuous data were expressed as a mean plus standard deviation (SD) when normally distributed and as a median with a range if not. The clinical and socio-demographic characteristics were compared with the Student *t*-test (for normally distributed data) or a Mann–Whitney U-test (for not normally distributed data) for continuous variables and with the chi square test for categorical variables. Multivariate logistic regression analysis was performed to identify factors associated with HRQoL. The odds ratio (OR) was used as a measure of association between the results of the questionnaires and the presence of a certain variable, adjusted then by the effect of the confusing variables. The agreement between the questionnaire and reports from the medical interview, obtained during the evaluation conducted with volunteers, was evaluated with the κ statistic, which is a measure of the agreement between two observers or tests. Values range from 0 and 1, where 1 is perfect agreement and 0 is what would be expected by chance. A value of *p* < 0.05 was considered statistically significant. The data were analyzed using the PASW statistic 18.0 software (IBM SPSS Statistics, Chicago, IL, USA).

## 3. Results

### 3.1. Demographic and Clinical Characteristics of Study Population

During the study period, 202 patients with IBD (29% CD and 71% UC) who met the inclusion and exclusion criteria were enrolled. All the participants were native residents. Less than one third of the enrolled patients showed comorbidities which required medication, mainly represented by hypertension, dyslipidaemia, diabetes, and thyroid diseases. All these conditions were under therapy control and did not represent a potential confounding factor for the investigation. Pediatric onset, surgery occurrence for IBD, and biologic use were more frequent in CD patients. Demographic and clinical characteristics of the study population are reported in Table 1.

**Figure 1:**
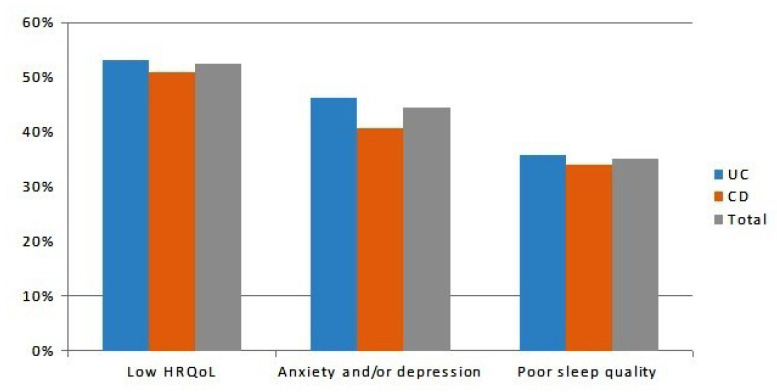
Percentages of the 202 inactive inflammatory bowel disease (IBD) patients (*n* = 143 UC and *n* = 59 CD) with a low health-related quality of life (HRQoL), anxiety and/or depression status, and poor sleep quality. UC: ulcerative colitis; CD: Crohn´s disease.

**Figure 2:**
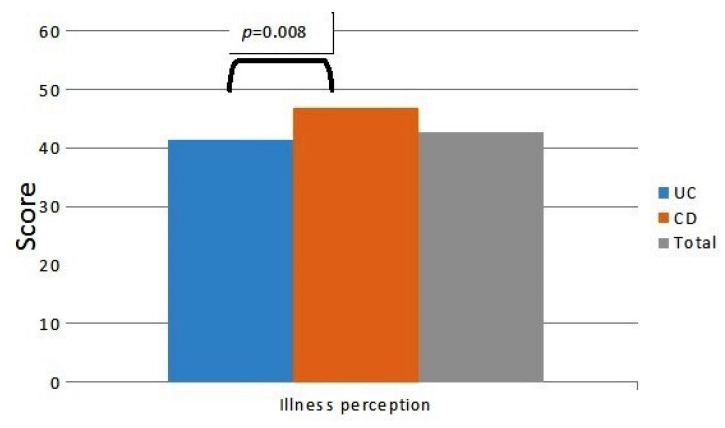
Illness perception overall score (mean score 42.8 ± 14.3) in the 202 IBD patients in remission (*n* = 143 UC and *n* = 59 CD), as assessed by Brief Illness Perception Questionnaire.

Slightly more than half (*n* = 106, 52%) of patients showed a low HRQoL (S-IBDQ < 50), with no difference between CD and UC (*n* = 30, 51% vs. *n* = 76, 53%, *p* = 0.56, respectively, Figure 1). Less than half (*n* = 90, 45%) of the patients reported anxiety and/or depression spectrum disorders (*n* = 24, 41% vs. *n* = 66, 46% among CD and UC patients, *p* = 0.14, respectively, Figure 1). Poor sleep quality was found in 71 (35%) patients, of which 20 (34%) had CD and 51 (36%) had UC, respectively (*p* = 0.84, Figure 1). According to the B-IPQ, there was a high perception of the disease, with a mean score of 42.8 ± 14.3 in the overall population (46.9 ± 12.1 in CD vs. 41.5 ± 14.8 in UC patients, *p* = 0.008, respectively, Figure 2).

### 3.2. Factors Associated with Low Quality of Life

As shown in Table 2, suffering from UC (*p* = 0.037), a previous IBD-related surgery experience (*p* = 0.021), a long-standing disease (*p* = 0.010), poor quality of sleep (*p* = 0.014), a higher perception of the disease (*p* = 0.006), and the presence of anxiety/depression (*p* = 0.042) were independently associated with a low HRQoL. At only univariate analysis, a pediatric onset of the disease was associated with a low HRQoL (*p* = 0.038), but it no longer persisted when adjusted for the other variables. All the remaining clinical and demographic variables did not show any significant differences between patients with low and normal HRQoL.

### 3.3. Impact on Working and Social Activities

The majority of patients (126, 62%) reported that the disease hindered their working activities. However, no differences were found according to type of disease and age, while females were more impaired than males (Table 3). IBD is associated with a negative impact on social activities in 149 (74%) patients, with a trend for younger individuals to be more impaired due to the disease. No differences in limitation of social activities were found according to type of disease or gender (Table 4).

### 3.4. Reality of Health-Related Quality of Life and Perceived Unmet Needs

**Figure 3:**
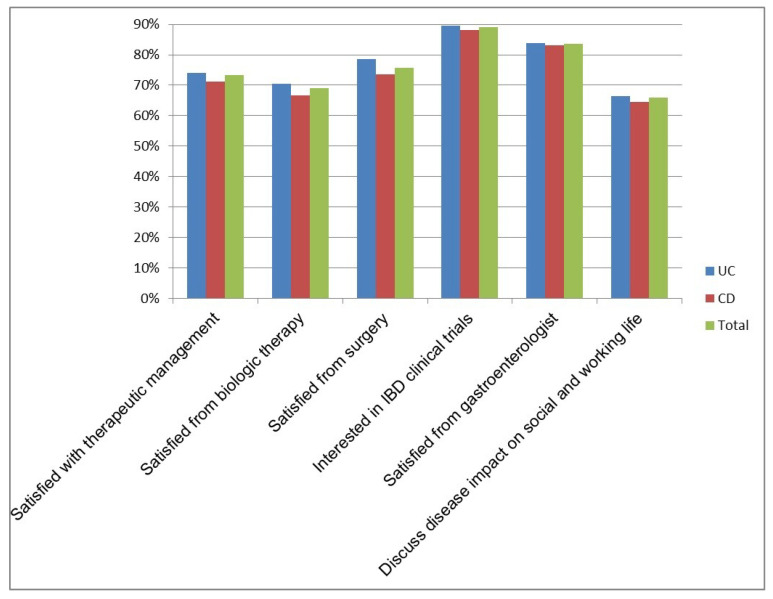
Reality of health-related quality of life and unmet needs (percentages of affirmative responses) among the 202 IBD patients in remission (*n* = 143 UC and *n* = 59 CD), as assessed by specific items [20].

One hundred and forty-eight patients (73%) were satisfied with their current therapeutic management. Of the 84 (42%) patients who were biologic-experienced, 58 (69%) were satisfied with the results obtained. Of the 33 (16%) patients who underwent surgery, 25 (76%) considered the approach useful. Regarding the possibility of trying a new drug for IBD, 180 (89%) patients were interested in clinical trial participation and would consider this chance for an improvement in their symptoms. Satisfaction with the gastroenterologist’s professional skills was expressed by 169 (84%) patients, but only 133 (66%) stated that the impact of their IBD on social and working activities was discussed with the gastroenterologist during a routine visit. No differences in the above-mentioned features were found between CD and UC patients (Figure 3).

## 4. Discussion

Despite continuous improvements in medical and surgical therapies, the impact of IBD on the patients’ HRQoL remains significant [21]. Through a cross-sectional study involving a series of 202 IBD patients in clinical remission from a single center of Southern Italy, we documented that 52% claimed a low HRQoL as assessed by S-IBDQ. As we have not considered a control group, the present results can only be set into context with already published data. Similarly to other studies [22,23], there were no differences in HRQoL between patients with CD and UC, although some reports suggest a more pronounced negative effect on HRQoL in CD patients due to the particularly severe psychosocial dysfunction [24]. Our findings are in keeping with results from a recent systematic review and meta-analyses showing that HRQoL for individuals with IBD is poorer than for healthy individuals [25]. Our study was able to show that, despite being in remission, patients reported a reduced HRQoL. As regression analysis confirmed, this was associated with previous surgery for IBD and a long-lasting disease, thus indicating that past experiences were considered by the patients. Consequently, it appears that patients evaluate HRQoL not only with regards to the current status of the disease. A large European observational study, which included a population of 1560 IBD patients from 31 medical centers, demonstrated that the majority of patients display a diminished HRQoL at both diagnosis and follow-up, as assessed by a S-IBDQ total score lower than 50 [26]. We chose the <50 cut off for low S-IBDQ scores in order to obtain results that are more representative of reality. Indeed, when a score of SIBDQ < 60 was used, the percentage of IBD patients with impaired HRQoL was definitely higher, ranging from 80% to 100% [27,28].

Mood disorders such as anxiety or depression were present in 45% of our patients. A comprehensive systematic review on the prevalence of anxiety and depression in IBD showed that anxiety disorders and anxiety symptoms are present in 21% and 35% of patients, respectively, while the prevalence of depressive disorder and depressive symptoms is 15% and 22%, respectively [29]. This association could be explained by the presence of fears and stress related to physical pain, a number of diagnostic tests, medical appointments, and frequent hospitalization. Nevertheless, a significant proportion of IBD patients with psychiatric disorders remains undiagnosed and untreated [30]. Recently, Kubesch A. et al. conducted an online survey in a German cohort of unselected IBD patients, focusing on overall emotional stress and the potential related factors, by using a questionnaire developed in cooperation with IBD patients. Results showed the presence of significant emotional stress but only 30% of the patients were in contact with the social care system and the corresponding support [31]. Factors such as sleep disorders, asthenia, and loss of appetite and weight, which often accompany the active course of IBD, could contribute to the onset or the worsening of pre-existing mood disorders [32]. Accordingly, poor quality of sleep was reported by 35% of patients, without distinction between CD and UC. Various studies have shown that IBD patients, even in the phase of disease remission, had significantly prolonged sleep latency, sleep fragmentation, and reduction of daytime energy [33]. Habibi F. et al. reported that 44% of IBD patients display poor sleep quality [34]. Graff L.A. et al. identified sleep disturbance in 77% of patients with active disease and in 49% with inactive disease [35]. An enhanced perception of the disease was present in our cohort of IBD patients which took part in the survey, with a significant increase in CD patients compared to UC patients. Illness perception may have a strong impact on both coping and treatment adherence; therefore, it is fundamental to consider this aspect in the management of IBD patients. Coping strategies are psychological and behavioral adaptations applied by the patient to improve the outcome of stressful situations and it has been demonstrated that maladaptive coping is associated with poor clinical outcome in IBD patients [36]. This suggests the importance of the continuous need for information and educational support in IBD patients, in order to provide them with the correct coping strategies. Especially as regards the youngest IBD patients, illness perception has been proposed as a target for psychological intervention [37].

Although HRQoL in IBD patients has been largely investigated in the last few years, studied often are focused on a precise item, such as mood disorder, working life, sleep disturbance, or illness perception [11,13,37]. A strength of our study is that we investigated at the same time HRQoL, anxiety/depression status, illness perception, and sleep quality, along with the clinical and demographic characteristics of the patients. Indeed, we sought to identify the potential burden of these items, which may relate to poorer quality of life in IBD. Through multivariate logistic regression analysis, suffering from UC rather than CD was significantly associated with a low HRQoL. This result did not appear in the univariate analysis and could be assumed to relate to the longer disease duration in UC patients along with the lower number of CD patients. Data on the association between HRQoL and type and duration of IBD are conflicting, as some authors suggest that patients with CD and short disease duration have the lowest HRQoL [22], while others did not find any predictive role of disease duration and type [38,39].

The impact of previous surgery due to medically refractory disease or disease complications has been considered as a factor influencing HRQoL. A lower HRQoL was documented in patients who experienced surgery for their disease compared to those free of surgery, and these data are supported by results obtained in the Italian population [40]. In addition, European data demonstrated that patients with UC who underwent surgery had significantly poorer disease-specific and generic quality of life during follow-up when compared to other UC patients who did not experience surgery [26]. On the contrary, Ponsioen et al. reported the beneficial effects of surgery on HRQoL in patients with CD [41]. Considering the role of sleep disturbance in impairing the host defense mechanisms and enhancing the effect of the inflammatory response [33], we investigated the presence of sleep disturbance and found that it was independently associated with a low HRQoL. This is not surprising as many reports have documented the presence of poor sleep quality in IBD patients, supporting the importance of considering quality of sleep as a factor in medical disease management [35].

An increased illness perception was found to be associated with lower HRQoL, although enrolled patients were in a quiescent phase of the disease. Illness perception as expressed by the patient though a self-administered questionnaire provides an important patient perspective on a disease that might not be captured by a clinical examination during a routine visit [19]. In general, patient-reported outcomes are considered as any report regarding the patient’s health condition that comes directly from the patient, without interpretation of the patient’s response by a clinician. The great value of patient-reported outcomes is that they include information regarding HRQoL, symptoms, satisfaction with care, adherence to medications, and perceived value of treatment, which have consequences for the patient’s overall picture [42]. The perception of disease has been recognized as a predictive factor of dimensions such as HRQoL, self-management of the disease, and adherence to medical therapy [10]. Epidemiological evaluations also suggest disconnections between what is relevant for the gastroenterologist (e.g., frequency of evacuations, bleeding, cessation of smoking, adequate treatment) and what is important for the patient (e.g., chronic fatigue, dyspepsia, emotional state, stress, and work disability), underlining how the patient’s perception of the disease is closely linked to their quality of life [43]. As expected, IBD patients displaying anxiety and/or depression symptoms were more likely to claim a low HRQoL. Anxiety-related and/or depression-related disorders strongly affect the quality of life of IBD patients, so much so that psychological symptoms should be considered when planning interventions to improve HRQoL in these patients [44]. Some studies, based on the use of the HADS questionnaire, supported the idea that keeping patients in remission has a positive impact on quality of life by reducing levels of depression. However, our results suggest that IBD patients experience concern and apprehension during periods of remission as well, probably due to the unpredictability of the course of the disease and the fear of complications [45]. Therefore, it is quite important to provide and maintain psychological care also for patients in remission, without underestimating this delicate issue [46].

More than half (62%) of participants reported that the disease represented an obstacle to their work performance. Several studies have shown that IBD strongly impacts work productivity [21,47]. Patients with IBD, significantly younger than those suffering from other chronic diseases, experience an unpleasant condition of work disability, with an increase in unemployment and sick leave and a consequent high socio-economic impact [48]. Social life was negatively influenced by the disease in 74% of the patients in our series and this agrees perfectly with current data showing that up to 75% of IBD patients claimed symptoms which interfere with their ability to enjoy leisure activities [47].

An interesting aspect of our survey was the investigation of patients’ satisfaction and their perceived unmet needs other than merely regarding clinical management. The majority of the interviewed patients (73%) were satisfied with their current therapy and so were patients who experienced biologic therapy (69%) and surgery approaches (76%). Healthcare professionals have become aware that patient satisfaction with their therapy is associated with benefits in clinical outcome, reduced overall costs, and improved quality of life [49]. Of note, most of the enrolled patients showed an interest in participating in clinical trials in order to try new therapeutic approaches, and this is consistent with the few reports currently available which consider the barriers to enrolling IBD patients in clinical trials [39,50]. Satisfaction with the assistance provided by the gastroenterologist was found in 84% of patients, confirming that quality of care is an essential issue to be addressed by clinicians [51]. The chronic relapsing nature of IBD requires a continuous interaction with the health system, including outpatient visits with specialists as well as hospital admissions. Patient satisfaction is considered to be an integral part of good HRQoL, so it is essential that medical staff assess patients’ satisfaction and expectations in providing healthcare [21].

Nevertheless, in our series, only 66% of the patients reported discussing the impact of their disease on their daily life during routine visits with their doctor, and this suggests that physician–patient interactions need to be improved. More work still needs to be done to better understand the relational problems that derive from the underlying disease, while the type of care should be more patient-centered [52]. Probably, more empathy should be demonstrated by clinicians in terms of the impact of IBD on work and quality of life, as physicians still are too focused on treating the disease, paying less attention to patients’ emotional status [53]. Indeed, it is recognized that certain issues are highly relevant to the patient but might be missed by the treating physician, due to the different perceptions between physician and patient. Although treatment practices can vary in individual countries due to the different structures of health services and resources, both patients’ and physicians’ perceptions should be considered when guiding appropriate management and therapies, in order to improve patient care [54].

## 5. Conclusions

In a series of IBD patients in clinical remission from Southern Italy, psychosocial elements such as sleep disturbance, anxiety and/or depression, and increased perception of the disease were independent factors associated with low HRQoL. Patients complained of a consequent severe disadvantage in their social and working lives, even during the quiescent phase of the disease. Notwithstanding the general satisfaction with standard quality of care, physician–patient interactions were limited according to patients’ perceptions. Patient-reported outcomes and patients’ disease perceptions should be taken into account by the treating physicians in order to positively affect clinical outcomes.

## Figures and Tables

**Table 1:** Demographic and clinical characteristics of the 202 patients with inactive inflammatory bowel disease (IBD).

Variable	CD *n* = 59	UC *n* = 143	Overall *n* = 202
Gender, *n* (%)			
Female	25 (42)	67 (47)	92 (46)
Male	34 (58)	76 (53)	110 (54)
Age, median (range), years	47 (19–80)	50 (17–82)	48 (17–82)
Pediatric onset, *n* (%) *	10 (23)	15 (14)	25 (17)
BMI, mean ± SD, kg/m^2^	23 ± 2	24 ± 3	24 ± 3
BMI > 24.9, *n* (%)	12 (28)	42 (39)	54 (36)
Smoking, *n* (%)	13 (26)	38 (25)	51 (25)
Marriage/cohabiting, *n* (%)	25 (50)	77 (51)	102 (51)
High education, *n* (%)	36 (83)	80 (75)	116 (77)
IBD surgery, *n* (%) **	19 (38)	17 (11)	36 (18)
Disease duration, mean ± SD, years	13 ± 11	15 ± 10	14 ± 11
Steroid-dependance, *n* (%)	21 (42)	54 (35)	75 (37)
Biologic experienced, *n* (%) ***	25 (50)	45 (30)	70 (35)

Values are numbers (*n*), percentage (%), and mean ± SD as indicated. SD: standard deviation; UC: ulcerative colitis; CD: Crohn’s disease; BMI: body mass index. Means were compared with the use of a Student’s *t*-test when data were normally distributed and with a Mann–Whitney U-test when data were not normally distributed, and proportions were determined with the use of a chi-square test. * *p* = 0.02; ** *p* = 0.001; *** *p* = 0.009.

**Table 2:** Demographic and clinical characteristics of the 202 IBD patients in remission according to their health-related quality of life (HRQoL) status.

Variable	Low HRQoL *n* = 106	Normal HRQoL *n* = 96	*p-*Value	OR (95% CI) Adjusted ^a^	*p-*Value (MVA)
Gender, *n* (%) Female Male	51 (48.1) 55 (51.9)	41 (42.7) 55 (57.3)	0.441	0.641(0.319–1.286)	0.210
Age, median (range), years	47 (17–82)	43 (17–74)	0.299	0.977 (0.947–1.007)	0.128
Type of disease, *n* (%) UC CD	76 (53.2) 30 (50.8)	67 (46.8) 29 (49.2)	0.566	2.449 (1.057–5.675)	**0.037**
Pediatric onset, *n* (%)	18 (17)	7 (7.3)	**0.038**	0.380 (0.115–1.251)	0.111
Family history of IBD, *n* (%)	18 (20.5)	13 (18.1)	0.702	1.314 (0.734–3.103)	0.672
Overweight, *n* (%)	41 (38.7)	39 (40.6)	0.778	1.169 (0.568–2.407)	0.818
Smoking, *n* (%)	26 (24.5)	25 (26)	0.805	1.269 (0.560–2.875)	0.568
High education, *n* (%)	59 (55.7)	59 (61.5)	0.404	1.114 (0.527–2.356)	0.777
Marriage/cohabiting, *n* (%)	59 (55.7)	43 (44.8)	0.123	0.701 (0.309–1.593)	0.397
IBD surgery, *n* (%)	25 (23.6)	12 (12.5)	**0.042**	3.533 (1.218–10.204)	**0.021**
Disease duration, mean ± SD, years	15.42 ± 11.3	13.23 ± 8.4	0.142	2.974 (1.304–6.781)	**0.010**
Steroid-dependence, *n* (%)	44 (41.5)	31 (32.3)	0.176	0.932 (0.443–1.960)	0.852
Biologic experienced, *n* (%)	43 (40.6)	27 (28.1)	0.064	0.658 (0.292–1.482)	0.312
Poor sleep quality, *n* (%)	52 (49.1)	19 (19.9)	**0.001**	2.609 (1.211–0.618)	**0.014**
Illness perception score, mean ± SD	48.08 ± 11.5	37.13 ± 15	**0.001**	1.039 (1.011–1.067)	**0.006**
Anxiety/depression status, *n* (%)	65 (61.3)	27 (28.1)	**0.001**	2.145 (1.028–4.464)	**0.042**

Values are numbers (*n*), percentage (%), and mean ± SD as indicated. MVA: multivariate analysis; SD: standard deviation; UC: ulcerative colitis; CD: Crohn’s disease. Means were compared with the use of a Student’s *t*-test when data were normally distributed and with a Mann–Whitney U-test when data were not normally distributed, and proportions were determined with the use of a chi-square test. Odds ratio (ORs) with 95% confidence interval (CI) in brackets are given. Bold text indicates a statistically significant difference with a *p*-value less than 0.05. ^a^ All variables except age, disease duration, and illness perception entered MVA analysis as categorical variables.

**Table 3:** Impact of IBD on working activities in the 202 patients with inactive disease.

Variable	Impact on Work *n* = 126	No Impact on Work *n* = 76	*p-*Value
Gender, *n* (%) Female Male	66 (52) 60 (48)	26 (34) 50 (66)	**0.012**
Age, median (range), years	45 (17–82)	45 (17–80)	0.649
Type of disease, *n* (%) UC CD	92 (73) 34 (27)	51 (67) 25 (33)	0.371

Values are numbers (*n*), percentage (%), and mean ± SD as indicated. SD: standard deviation; UC: ulcerative colitis; CD: Crohn’s disease.

**Table 4:** Impact of IBD on social activities in the 202 patients with inactive disease.

Variable	Impact on Social Activities *n* = 149	No Impact on Social Activities *n* = 53	*p-*Value
Gender, *n* (%) Female Male	71 (48) 78 (52)	21 (40) 32 (60)	0.313
Age, median (range), years	43 (17–82)	52 (26–81)	0.07
Type of disease, *n* (%) UC CD	104 (70) 45 (30)	39 (74) 14 (26)	0.603

Values are numbers (*n*), percentage (%), and mean ± SD as indicated. SD: standard deviation; UC: ulcerative colitis; CD: Crohn’s disease.

## Supplementary Materials

The following are available online at https://www.mdpi.com/2077-0383/9/8/2416/s1, File S1: Questionnaire regarding the impact of the disease on the life of the patient.
