# Supplementary material for: The Reality of Patient-Reported Outcomes of Health-Related Quality of Life in an Italian Cohort of Patients with Inflammatory Bowel Disease: Results from a Cross-Sectional Study
Source: J Clin Med. 2020 Jul 28;9(8):2416. doi: 10.3390/jcm9082416 (PMC7464775; doi:10.3390/jcm9082416)
Supplement: Supplementary file 1 [file jcm-09-02416-s001.pdf]

Supplementary Material

Questionnaire regarding the impact of the disease on the life of the patient

Please answer with a X in the box as appropriate:

1) Do you believe that your intestinal disease affects a lot your working activities?
YES ☐ NO ☐

2) Do you believe that your intestinal disease affects a lot your social life?
YES ☐ NO ☐

3) Are you satisfied with your current therapy for your intestinal disease?
YES ☐ NO ☐

4) If you experienced biologic therapy for your intestinal disease, are you satisfied with it?
YES ☐ NO ☐

5) If you experienced surgery for your intestinal disease, are you satisfied with it?
YES ☐ NO ☐

6) Would you join a clinical trial testing a new drug for your intestinal disease?
YES ☐ NO ☐

7) Are you satisfied with your gastroenterologist as professional figure?
YES ☐ NO ☐

8) Do you discuss the impact of your intestinal disease on working activities and social life during routinely visit, in order to share with your gastroenterologist your perceptions and unmet needs?
YES ☐ NO ☐

9) In the last month, did you suffer from sleep disturbance?
YES ☐ NO ☐
